# Decision-makers’ acquaintance with the public’s priorities in health services

**DOI:** 10.1186/s13584-016-0081-8

**Published:** 2016-06-28

**Authors:** Giora Kaplan, Orna Baron-Epel

**Affiliations:** Psychosocial Aspects of Health, The Gertner Institute for Epidemiology and Health Policy Research, Sheba Medical Center, Ramat Gan, 52621 Israel; School of Public Health, Faculty of Social Welfare and Health Studies, University of Haifa, Haifa, Israel

**Keywords:** Health priorities, Consultation with the public, Health rationing, Decision-makers opinion, Public opinion

## Abstract

**Background:**

Decision makers often assume they know the public’s standpoints and see themselves as capable of representing them.

The aim of this study is to assess the level of acquaintance that senior decision-makers in the Israeli health system have concerning the priorities of the public in whose name they act.

**Methods:**

A phone survey was conducted with a representative population sample and face-to-face interviews were conducted with senior decision-makers.

**Results:**

The decision-makers did predict correctly the public’s desired level of government involvement in health care; but only some of them correctly predicted the public’s preferences on allocation of funds—to health versus other areas. They had difficulty foreseeing public priorities for allocating additional monies to health, and even greater difficulty ascertaining preferences of the public for their own health insurance.

**Conclusions:**

Government decision-making processes should include evidence about public preferences. The findings of this study indicate that decision makers need to be provided with reliable, systematic information on public preferences.

## Background

Making decisions on priority setting involves understanding the priority setting situation, the purpose or goal to be achieved, the available alternatives, the probable consequences of each alternative and the values to the decision maker on these probable consequences. Priority setting in a state of limited resources is an economic challenge, as well as a political conundrum. Politicians tend to avoid explicit rationing of social services, particularly health services, or tend to transfer responsibility to others (such as expert committees or HMOs) in order to avoid taking responsibility for unpopular choices [4].

Both priority setting and health care decisions related to rationing require making difficult choices between noble alternatives that may result in denying access to certain services. There is rarely a consensus among all those involved in the decision. It is even difficult to reach agreement on the principles that should guide the decision-making process and on their relative weight. It is essential that the decision-making process itself be seen as legitimate and fair, and that decisions made are justified with a logical and reasonable explanation [4].

Thus, priority setting in health care is complex. Professional knowledge and considerations are intertwined with ethical, legal, political and social considerations. Decision makers should be particularly interested in understanding the public’s priorities, both because in a democracy public policy should serve the public and because a policy that is not acceptable to the public will be more difficult to implement. Policymakers and politicians must find ways of gauging, interpreting, and maneuvering within or around public opinion.

Health policy decision makers do not ignore public opinion; however, they take public opinion into account only on certain issues. On other issues they still take a paternalistic stance. They seem to consider the public to be unable to understand or express an opinion on issues regarded by healthcare executives as being highly professional or complex. Even when such policymakers state that decision making should take into account the position of the public, they generally make a subjective assessment of public opinion which is neither systematically obtained nor reliable.

Many studies dealing with rationing and prioritization have demonstrated a difference between public opinion and the opinion of politicians and health professionals [1, 3, 9–12, 15, 17]. Susan Herbst [5] examined how state-level politicians and their staff “read” public opinion when polling data are sparse and they must construct the views of the public through other means. She found that political actors are largely unconcerned about the potential biases of these sources.

Our paper aims to examine the extent to which senior decision-makers in the Israeli health system are familiar with the priorities of the public in whose name they act.

## Methods

### Population survey

A telephone survey was conducted with a representative sample of the Israeli adult population aged 18 and over (*N* = 1225). The sample design as well as the collection of data was performed by The Cohen Institute for Public Opinion Research from Tel Aviv University. The phone interviews were conducted during June-August 2008 and were carried out in Hebrew, Arabic and Russian. The total response rate was 36 % [6, 7].

**Fig. 1:**
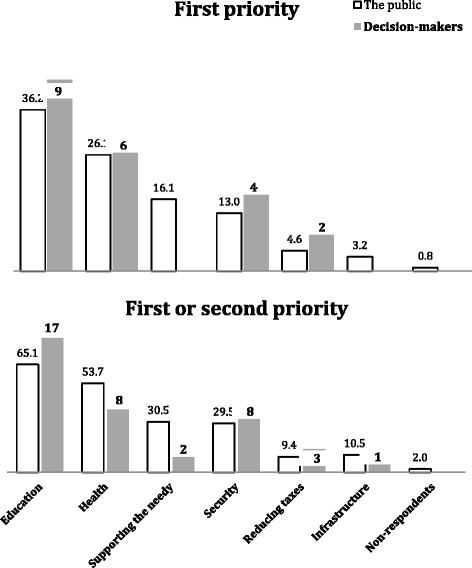
Decision-makers forecast of the public’s priority for receiving the extra budget

Regarding expectations from the government in healthcare, interviewees were asked to choose one of three levels of involvement (see response options in Fig. 1). The relative importance the population attributes to the health area was assessed by a question asking to which area interviewees would transfer an extra budget (see list of areas presented in Fig. 1).

The public’s priorities in health were assessed by asking interviewees to assume they were the Minister of Health and, as such, to rate on a four level scale, to what extent they would allocate an extra budget to each of the items presented to them. There were two versions of the questionnaire to overcome the limitation of the length of the questionnaire for a telephone survey. The items in one version of the questionnaire included: fertility treatments, cardiac rehabilitation, check-ups for early disease detection, mental health, subsidizing supplemental insurance for the poor and alternative medicine. The second version of the questionnaire included: nursing care for the frail elderly (i.e. community-based long-term care), dental health, programs for preventive medicine and health promotion, additional staff for primary clinics and building a new hospital. An additional question asked interviewees to indicate their first priority.

The public’s priority at the personal level was assessed by asking interviewees to rate the extent to which they would choose to include each one of the items in their personal complementary health insurance. From the previous lists a few items were deleted (subsidizing complementary insurance, building a new hospital, fertility treatments and adding clinic staff) and they were replaced by cosmetic surgery, obtaining a second opinion and hospitalization in a private hospital. Here again, interviewees were asked to rate, on a four level scale, each item and finally to choose their most important priority.

Data analyses were performed using the 9.13 release of SAS PC computer software. Chi-square tests was used in the univariate [perhaps: bi-variate?] analyses. Multivariable logistic regression analysis was performed including an assessment of which independent variables were significant at a 20 % level.

### Interviews with decision makers

The sample included the most senior officials in the health system who are personally involved in national policy determination. Participants were recruited using the ‘snowball method’. A basic list was constructed of senior officials in the main institutions of the Israeli health system. During the interviews, they were asked who, in their opinion, should be included in the sample of senior decision makers. The final sample included 21 decision makers.

In the table below, respondents’ positions and affiliations are presented according to their position at the time of the interview. As expected, most of them held additional senior positions in the health system in the past, and some of these previous roles are mentioned in brackets.

**Table:** 

Ministry of Health	Sick Funds
Director General (+3 former)	Director General (+1 former)
Deputy Director-General (+1 former)	Head of Community Services Division
Head of Medical and Health Services Administration (+1 former)	Medical Director
Head of Health Economics and Health Insurance Division	
Head of Public Health Services	Public Hospitals
Head of Medical Technologies and Infrastructure Administration	3 Director Generals
Head of Mental Health Services	
Legal Advisor	Private Hospitals
Ombudsman	1 Director General
Former Chairman of the Health Services Basket Committee	1 Chairman of the Board Managers
Israel Medical Association	Ministry of Finance
Secretary General and Legal Advisor	Deputy Director—Budget Division
	Health Referent (+1 former)

Data collection: All the personal interviews were conducted face-to-face by one the investigators. After an explanation of the study objectives was provided, an open question was asked on what should be, in the opinion of the interviewee, the level of the government’s involvement in health care. Following this question, the prioritization questions were presented to the interviewee and s/he was asked to predict how the public’s responses would be distributed between the different options presented.

## Results

Decision-makers were presented with the survey questionnaire and they were asked to forecast what they thought the public would answer to selected questions. Most (18 of 21) correctly predicted the distribution of the public’s answers regarding the desired level of the government’s involvement in health care (Table 1). However, less than half (9 of 21), correctly predicted that the highest support amongst the public would be education. Six thought that health would receive the highest support. Six predicted that public would prioritize investment in security and tax reduction, but only a small percent of the public gave first priority to these issues (Fig. 1).

**Table 1:** Decision-makers forecast of the public’s preferences for the government role in healthcare and the actual distribution of the public’s responses in the survey

The options presented	Decision-makers forecast	Distribution of the public’s responses
	# (%)	%^a^
1. The government provides healthcare for everybody and finances them from the taxes collected from the citizens. There are no HMOs, the government is the HMO of everybody.	2 (10)	35
Between options 1 and 2	3 (14)	
2. Health Care Organizations provide healthcare. The citizen pays health insurance to government and the government distributes the revenues among the HMOs	15 (71)	43
3. All is private. The citizen buys him/herself health insurance. The government only supervises and regulates health services and only funds the insurance for vulnerable populations.	0	18
Between 1 and 3	1 (5)	

^a^4 % non-respondents

When we asked for less specific answers from the decision makers, it was found that most (17 of 21) correctly predicted that the majority of the public would choose education for their first or second priority, but only a third of them predicted that over half of the sample would choose health as their first or second priority.

**Fig. 2:**
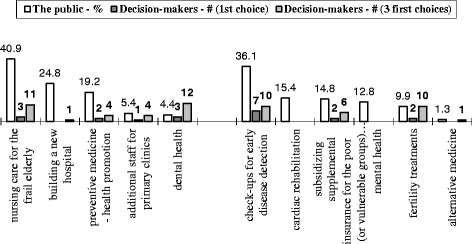
Decision-makers forecast of the public’s priority of the area in healthcare to be awarded with the extra budget

When the officials were asked to predict which area in the health arena the public would prioritize for receipt of additional budget, there was a lack of consensus amongst them (Fig. 2). When we required less precision, just predicting the area the public mentioned in the first three places, half estimated correctly that a significant percentage of the public would choose care for the elderly and tests for early detection of disease, even though only 3 and 7 of the decision makers mentioned these areas in the first place. Building a new hospital and cardio-rehabilitation were prioritized by a significant percentage of the public, but were not mentioned at all by the executives. The decision makers predicted that the public would give priority to dental and fertility treatments, but they were actually selected by only a small percentage of the public.

**Fig. 3:**
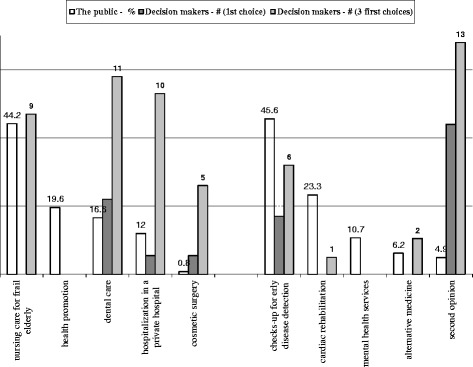
Decision-makers forecast of the public’s priority of the area in healthcare to be included in their personal health insurance

Nineteen decision-makers provided a forecast of the public’s priorities in their personal insurance. In this area, they had more difficulty predicting the preferences of the public (Fig. 3). The two issues that had received the highest support amongst the public were early detection of disease and nursing care. However, only 6 of the 19 decision makers predicted that the former would be prioritized by the public, and only three mentioned this as first priority. Only 9 mentioned the latter, and none mentioned this as the first priority.

Decision makers did not foresee at all the two areas that received the next highest prioritization from the public—health promotion and cardiac rehabilitation. Rather, decisionmakers predicted that the public had prioritized second opinions, dental care and hospitalization in a private hospital. Second medical opinion, mentioned the least by the public of all the areas presented, was stated as a first place consideration by eight executives.

**Fig. 4:**
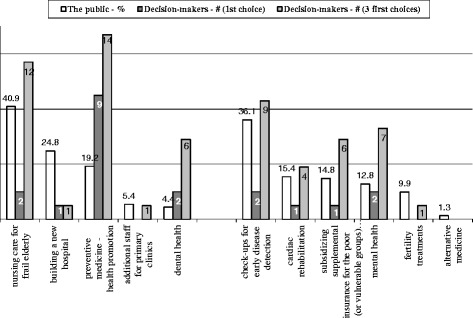
Decision-makers’ priorities* of the area in healthcare to be awarded with the extra budget. * One official did not rank his 3 priorities so that the first priority includes only 20 decision makers

Decision makers were also asked to express their personal priorities about the issues presented in the questionnaire. Figure 4 shows that there was consensus between them only regarding the importance of prevention projects and health promotion; 14 participants indicated them as being among the three most important areas and 9 even ranked them in first place. Nursing care and tests for the early detection of disease, ranked first amongst the public, were also placed among the more important issues by many executives.

## Discussion and conclusions

In this survey, the most senior decision makers in the Israeli health system, who are not elected political figures, were interviewed independently from a survey of the public. The accuracy of their assessment of public opinion was mixed; and there were some important inaccuracies. For example, decision makers perceive the public as preferring what they call “indulgences” (second opinion, hospitalization in a private hospital), while the public participating in the survey expressed a strong preference for nursing care and preventative care.

In this study we did not investigate the sources decision makers used in order to develop their perceptions of public opinion. We believe that decision-makers widely use non-systematic sources in order to develop their own image of the public opinion. Studies in other areas have provided useful information on this subject [5, 8, 13, 14].

Rosner [14] found that political actors are ‘hunter gatherers’ for information on public opinion. He found reading public opinion to be a complex undertaking in which people ‘thin slice’ the world around them and creatively construct opinion from a range of sources for both immediate and predictive uses. Herbst’s findings [5] indicate that political actors often confuse information in the media sources and interest group communications with general public opinion. Brown, in her study on public opinion and penal policy [2], found that political actors in New York State assume that they, as individuals, are able to remain independent from undue influence of media-generated and/or hysterical public concerns and can tap into what average people really care about. They assume, however, that other political actors are swayed by sensationalized public opinion, often in the form of media coverage.

Kull and Ramsay [9] identified two key dynamics that could well contribute to policymakers misreading the public: a failure to seek out information about the public and a tendency to assume that the vocal public is representative of the general public. Sometimes decision makers mistakenly interpret the position of the media or the views of interest groups as representing public opinion, and sometimes they incorrectly attribute these attitudes to the majority of the public [8–10].

There are several ways by which policymakers may misperceive public sentiments: Herbst’s findings [5] show that policymakers may do it because they received flawed inputs; whereas, Cook et al [11] suggest that policymakers invoke public opinion to support positions they already hold; and Kull and Ramsey [10] suggest that deeply held assumptions about public views are simply taken for granted, even when are at odds with the facts.

To our knowledge, ours is the first attempt in the health domain to test the ability of decision makers to predict the public’s preferences. At the present time, in Israel, even though many policy makers in health state the importance they attach to giving the public a chance to express their opinion, there is no real pressure to consult and listen to the public. Only a few attempts have been made to consult with the public, and little use has been made of the findings from public opinion polls.

The findings of this study indicate that decision makers do not have reliable, systematic information on public preferences. Some policy makers and even some policy analysts believe that polls tell us very little about public policy and are unlikely to be improved enough to help with policy choices [16].

A variety of mechanisms can be used to reveal the public’s opinion: from non-systematic methods such as asking the public to express their opinion through a web site, to representative population surveys, to a variety of techniques of managed deliberations such as focus groups, nominal groups, citizen’s parliament or citizen’s jury. A combination of a survey with group discussions may produce particularly useful information, since this facilitates deeper understanding of the public’s opinions after they were supplied with relevant information and understanding of the issue. The accompanying survey allows for systematic evaluation of the extent of support for various opinions, both in the general population and for key population sub-groups.

We strongly believe that it is necessary to raise important policy issues and integrate them into public discourse. We also believe that it is the responsibility of government to inform and explain to the public the complexity of the issues. We want to emphasize that we do not advocate the use of polls or referendums to determine health policy decisions. The rationing of health care requires evaluation of the effectiveness of diverse medical treatments, cost-benefit calculations and price setting of services. These are tasks that are not appropriate for a survey in the general public.

Some policy analysts claim that public opinion expressed in polls cannot inform policy choice, because that process requires attention to tradeoffs among values, second-best possibilities, and unexpected risks [16]. However, decision makers, when determining priorities between different services, are influenced not only by effectiveness and efficiency data but also by personal standpoints which they believe to be important. Therefore, it seems not just reasonable, but important, that they factor into the decision-making process accurate information on the value the public places on those services.

It is the public that will ultimately be affected by policy. Consultation and dialogue with the public can only strengthen the legitimacy of the decision-making process and its acceptance by the public. Even when the final policy decision may be contrary to popular public opinion, being acquainted with the views of the public enables decision-makers to prepare an effectively focused explanation or marketing campaign. Ironically, even the policy makers who least value public opinion, stand to gain from having obtained it.
